# HEALTHCARE UTILIZATION IN OLDER ADULTS (≥70 YEARS) UNDERGOING CELLULAR THERAPY FOR HEMATOLOGIC MALIGNANCIES

**DOI:** 10.1093/geroni/igac059.2536

**Published:** 2022-12-20

**Authors:** Christina Snider, Hina Budhwani, Melissa Logue, Parul Goyal, Kelly Pugh, Harvey Murff, Adetola Kassim, Reena Jayani

**Affiliations:** Vanderbilt University Medical Center, Nashville, Tennessee, United States; Vanderbilt University Medical Center, Nashville, Tennessee, United States; Vanderbilt University Medical Center, Nashville, Tennessee, United States; Vanderbilt University Medical Center, Nashville, Tennessee, United States; Vanderbilt University Medical Center, Nashville, Tennessee, United States; Vanderbilt University Medical Center, Nashville, Tennessee, United States; Vanderbilt University Medical Center, Nashville, Tennessee, United States; Vanderbilt University Medical Center, Nashville, Tennessee, United States

## Abstract

Increasingly, older adults are receiving hematopoietic cell transplant (HCT) and chimeric antigen receptor T-cell therapy (CAR-T), intensive therapies for treatment of hematologic cancers which typically require prolonged hospital admissions. Older adults are at high risk of increased healthcare utilization and complications of prolonged hospitalization [Mudge, J Am Geriatr Soc, 2019]. We identified patients age ≥70 years who received HCT or CAR-T in a primary outpatient transplant program at Vanderbilt University Medical Center between 1/1/19 and 12/31/20. Healthcare utilization, including all visits and admissions, was captured from the start of conditioning chemotherapy through early post-therapy. Thirty-eight patients met inclusion criteria; 26 (68%) received autologous HCT (autoHCT), 7 (18%) allogenic HCT (alloHCT), and 5 (13%) CAR-T. Twenty-four patients (63%) had high HCT-Comorbidity Index (HCT-CI). Eighteen (69%) autoHCT, 6 (86%) alloHCT, and no CAR-T patients had at least one unplanned admission. The median number of total hospital days (LOS) was 7.5 (2-14), 8 (4-62), and 9 (7-9) days, respectively. One-year mortality was 12% (3) in autoHCT, 43% (3) in alloHCT, and 0% in CAR-T. Low performance status and high HCT-CI did not correlate with LOS (p=0.58 and p=0.16, respectively) or number of outpatient visits (p=1, p=0.19). In conclusion, most patients who received auto- or alloHCT in a planned primary outpatient setting experienced at least one unplanned admission. LOS duration varied widely with shorter LOS among autoHCT patients. Further research is needed to identify factors among older adults (≥70 years) at risk of increased healthcare utilization during HCT or CAR-T.

